# Amputation of Part of the Tongue for Carcinomatous Disease

**Published:** 1873-06

**Authors:** 


### ARTICLE VI.

### *Amputation of Part of the Tongue for Carcinomatous Disease.*

Upon looking at this patient, (thirty-eight years of age,) you see at a glance that he is a sick man; his face is sallow and his frame is emaciated. He states that since he first began to be sick, nine months ago, he has lost over forty pounds in weight. The quantity of urine voided is normal in quantity and quality. His bowels are open, he has no flatulence, indigestion nor unusual thirst; his appetite is good, but he cannot eat; his hands and feet are warm; he never had intermittent fever or night-sweats, but he has a trouble of his tongue, which commenced four weeks ago, in which he experiences constant burning; at times lancinating pain, which seriously interferes with mastication and deglutition. As he protrudes the tongue, you notice a surface continuously ulcerated, principally on the left side of the median line, extending from the tip a considerable distance back. I can find no enlarged lymphatic glands under the jaw. His diet is necessarily confined to the blandest articles, and such as require no mastication—such as milk, beef-tea and milk punch. His face is not pallid or livid exactly, but has a sallow, cadaverous appearance peculiar to the cancerous cachexia.

This may be either of two affections—syphilis or epithelioma. A man may have a chancre on his lip or tongue by inoculation from a mucous patch, or directly from virus accidentally carried by the finger, or an ulcer may exist here as the result of constitutional syphilis. In such a case however, the ulcers have well-defined edges, generally with a foul, irregular base, and are not single, but appear in scattered patches; they are not confined to the tongue usually, but co-exist on the fauces, uvula and pharynx. There would also be evidences of specific disease in other portions of the body, and a distinct history of inoculation, neither of which exists in the present case.

Therefore, by exclusion, and from the appearance, hardness, and peculiar pain, I would consider this epithelial cancer, or cancroid, as it was termed by Prof. Miller, of Edinburgh; or, properly, scirrhus, modified by the structure involved. This generally comes without assignable cause. It has been attributed to smoking a short pipe, with the hot stem constantly against the tongue; but this is unsustainable by proof; the disease in some cases occurring in patients who never smoked, while thousands of others escape who are inveterate smokers. Encephaloid disease of the tongue is extremely rare, while colloid and melanosis in this situation are unknown. This disease is most common after the age of fifty, and in the male sex. It commences as a little sore, papule, or crevice in the tongue; it then gradually spreads and ulcerates, and is the seat of the characteristic pain, which is generally worse at night. The surrounding part is hard, from infiltration of plastic lymph and cancer cells, and the organ can no longer be used in mastication on account of its stiffness and the pain produced by moving it. The disease finally extends to the gums, the teeth fall out, the submaxillary glands enlarge, and the system sinks under the exhaustion from the cachexia which becomes established, and from impaired nutrition. The fatal result generally supervenes within a year or eighteen months from the onset of the disease.

The only operation likely to afford any relief would be excision of the affected part, not in the hope of cure, but to palliate the symptoms. But he is not in a condition to bear such interference at present; his general health must first be improved by relieving his pain and allowing him to sleep, by giving him gr. xxx. of chloral at night. He shall have plenty of soft, nourishing food, such as milk-punch, animal broths, soft boiled eggs, etc., with daily open air exercise, and may wash his mouth frequently with dilute solution of permanganate of potassa. It is important that the patient should not swallow the secretions, as pyæmia might result. I have seen this occur in two cases—in one after removal of a polypus, and in the other from plugging the posterior nares.

November 20, (one month later.)—The patient's health is much improved, and he returns for operation. The ulcer is now rapidly spreading beyond the median line of the tongue, and also on the under surface. Ablation, or excision of the tongue, is not required in this case; removal of the offending part is all that will be attempted. This should not be done with the knife, as there would be severe, perhaps uncontrollable, hemorrhage from the vessels, which are abnormally increased by the disease. This is one of the operations to which the ecraseur is eminently applicable, as it crushes and lacerates the vessels, producing rapid coagulation of the blood and diminishing hemorrhage. Before applying the chain of the instrument, the tongue is transfixed by three stout steel pins at the line where you wish to amputate. The ecraseur is then applied and the diseased mass removed. The lingual artery and a few small vessels required the ligature; the capillary oozing was checked by ice. The operation was performed without an anæsthetic, at the patient's request. An anodyne will be given immediately, and the former treatment continued, to which shall be added quinine and iron.

[The patient was subsequently shown at the clinic, and reported himself as being much more comfortable. He made a good recovery from the operation, and there was no secondary hemorrhage. He was then able to speak so as to be understood, although half of the anterior portion of the tongue had been removed transversely. He afterwards complained of a severe sore throat, and within two months from the operation, died from debility. No examination could be obtained.]—*Surg. Clinic of Prof. Gross.—Medical Times.*

